# Low-energy Ar^+^ and N^+^ ion beam induced chemical vapor deposition using hexamethyldisilazane for the formation of nitrogen containing SiC and carbon containing SiN films

**DOI:** 10.1371/journal.pone.0259216

**Published:** 2021-10-27

**Authors:** Satoru Yoshimura, Satoshi Sugimoto, Takae Takeuchi, Kensuke Murai, Masato Kiuchi

**Affiliations:** 1 Center for Atomic and Molecular Technologies, Graduate School of Engineering, Osaka University, Suita, Osaka, Japan; 2 Department of Chemistry, Biology and Environmental Science, Faculty of Science, Nara Women’s University, Nara, Nara, Japan; 3 National Institute of Advanced Industrial Science and Technology (AIST), Ikeda, Osaka, Japan; Nazarbayev University, KAZAKHSTAN

## Abstract

We proposed an experimental methodology for producing films on substrates with an ion beam induced chemical vapor deposition (IBICVD) method using hexamethyldisilazane (HMDS) as a source material. In this study, both HMDS and ion beam were simultaneously injected onto a Si substrate. We selected Ar^+^ and N^+^ as the ion beam. The energy of the ion beam was 101 eV. Temperature of the Si substrate was set at 540 °C. After the experiments, films were found to be deposited on the substrates. The films were then analyzed by Fourier transform infrared (FTIR) spectroscopy, stylus profilometer, X-ray diffraction, atomic force microscopy, and X-ray photoelectron spectroscopy (XPS). The FTIR and XPS results showed that silicon carbide films containing small amount of nitrogen were formed when Ar^+^ ions were injected in conjunction with HMDS. On the other hand, in the cases of N^+^ ion beam irradiation, silicon nitride films involving small amount of carbon were formed. It was noted that no film deposition was observed when HMDS alone was supplied to the substrates without any ion beam injections.

## Introduction

Silicon carbide (SiC) recently receives wide-spread attention of its superior characteristics and is used as a semiconductor material [1,2], while silicon nitride (SiN) has been similarly interested in its practical applicability in surface passivation [3]. In most cases, SiC films were produced with the chemical vapor deposition (CVD) method using silane and propane as source gas [4]. On the other hand, SiN films could be produced using silane and ammonia [5].

An ion beam induced chemical vapor deposition (IBICVD) method has long been recognized to be useful for the preparation of various oxide films such as TiO_2_ [6,7], Al_2_O_3_ [7], and ZrO_2_ [8]. It is conceivable that the practical application of the IBICVD method to topics other than the oxide film formation would be important and significant subjects. At present, the IBICVD method is attracting much attention for the fabrications of three-dimensional nanostructures [9] and ferromagnetic materials [10].

Recently, the IBICVD method is found to be of use for the formation of SiC films. Matsutani *et al*. used hexamethyldisilane [(CH_3_)_3_SiSi(CH_3_)_3_] as a source material of IBICVD experiments [11]. In their experiments, Ar ion beams were injected to Si substrates in conjunction with the gas flow of hexamethyldisilane. They found that SiC films were formed on the substrates due to the dissociation of hexamethyldisilane by the Ar ion impact. Matsutani *et al*. also confirmed that silicon dioxide films could be deposited with the IBICVD method using hexamethyldisiloxane [(CH_3_)_3_SiOSi(CH_3_)_3_] [12]. Subsequently, Yoshimura *et al*. succeeded to deposit SiC films with the IBICVD method using methylsilane [13].

Meanwhile, hexamethyldisilazane [HMDS, (CH_3_)_3_SiNHSi(CH_3_)_3_] has long been used in various scientific and technological studies. For examples, HMDS is available for the preparation of various biological specimens for microscopy [14,15], chemical syntheses [16–20], and the formation of SiN [21–23] and silicon carbonitride (SiCN) [24–30] films. HMDS has also been shown to be available for the deposition of SiC [31,32] and diamond-like carbon (DLC) [33].

The HMDS molecule has a chemical structure which is similar to hexamethyldisilane and hexamethyldisiloxane. Nevertheless, film deposition experiments with the IBICVD method using HMDS have not been carried out yet.

In this study, we tried to deposit films with the IBICVD method by injecting both ion beam and HMDS to a Si substrate. Both Ar^+^ and N^+^ were selected as the injecting ions. We examined the mass and energy distributions of the injecting ions prior to the IBICVD film deposition experiments, and after the IBICVD experiments, we assessed deposited films by Fourier transform infrared (FTIR) spectroscopy, stylus profilometer, X-ray diffraction (XRD), atomic force microscopy (AFM), and X-ray photoelectron spectroscopy (XPS).

## Materials and methods

This study was carried out with an ion beam system (ULVAC) [34]. The ion source of the system was a Bernas-type source [35]. In this study, pure Ar gas or pure N_2_ gas was used as a source gas for the Ar^+^ or N^+^ ion beam production. Ar^+^ or N^+^ ions produced in the ion source were extracted and accelerated by a high voltage of -15 kV. Then, the ions were mass-selected by a mass selector to remove impurity ions. The mass-selected Ar^+^ or N^+^ ion beams were finally decelerated to 100 eV and then led to the processing chamber. The base vacuum in the processing chamber is approximately 1x10^-6^ Pa. The mass and energy of the ion beams can be determined by a plasma process monitor PPM-421 (balzers).

**Fig 1:**
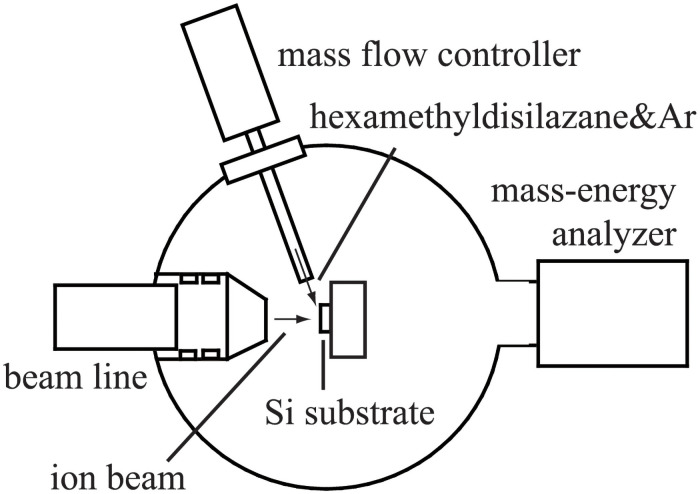
A schematic drawing of the processing chamber of the ion beam system.

A schematic diagram of the IBICVD equipment in the processing chamber of the ion beam system is shown in Fig 1. An untreated Si substrate (15x15 mm) was set on the substrate holder in the processing chamber (Fig 1). The energy of the ion beam was set at a low level (100 eV) to avoid significant damages of the deposited films by the ion injection. The incident angle of the ion beam was set to be normal to the substrate surface. Liquid HMDS in a container was bubbled using Ar gas and the resulting mixed gas of HMDS and Ar was supplied onto the substrate surface at a flow rate of 0.7 sccm through a stainless-steel tube connected to a mass flow controller (Fig 1). The gas pressure in the processing chamber was 1x10^-3^ Pa during the deposition experiment. The temperature of the Si substrate during the deposition experiment was set at 540 °C.

After the IBICVD experiments, we assessed deposited films by FTIR spectroscopy (FT/IR-410, Jasco), stylus profilometer (P-15, KLA-Tencor), XRD (RINT2200, RIGAKU), AFM (JSTM-4200D, Jeol), and XPS (AXIS-165x, Kratos).

## Results

### Production of Ar^+^ and N^+^ ion beams

**Fig 2:**
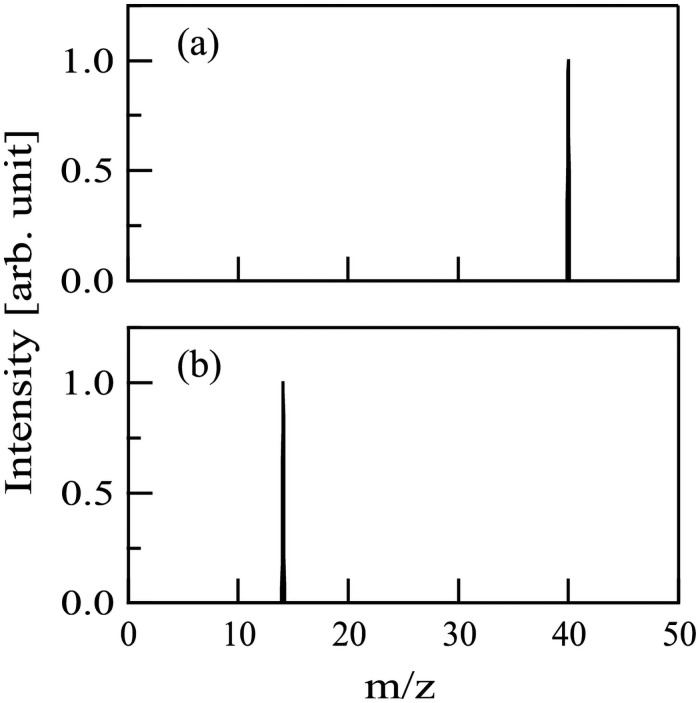
The mass spectra of (a) Ar^+^ and (b) N^+^ ion beams.

Before the IBICVD film deposition experiments, the mass spectrum of the ion beam was measured with PPM-421. It was found that only a single peak appeared at the mass number of 40 [Fig 2a] or 14 [Fig 2b], suggesting the presence of pure Ar^+^ or N^+^ ions without impurity ions.

**Fig 3:**
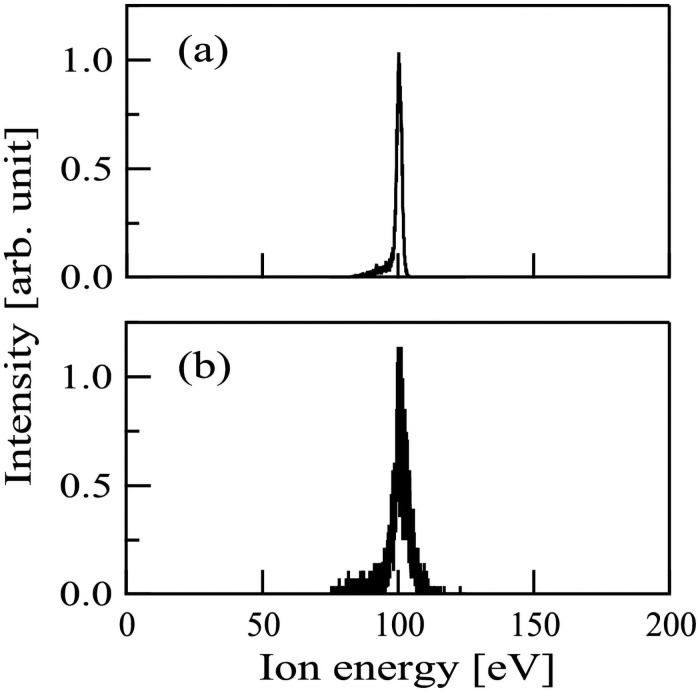
The energy spectra of (a) Ar^+^ and (b) N^+^ ion beams.

The ion energy spectra of Ar^+^ and N^+^ ion beams were also measured with PPM-421, as shown in Fig 3. Fig 3a and 3b show that the peak energies of (a) Ar^+^ and (b) N^+^ ion beams were 101 eV. Fig 3a and 3b also show that the ion beams used in this study were nearly monochromatic in energy distribution.

**Fig 4:**
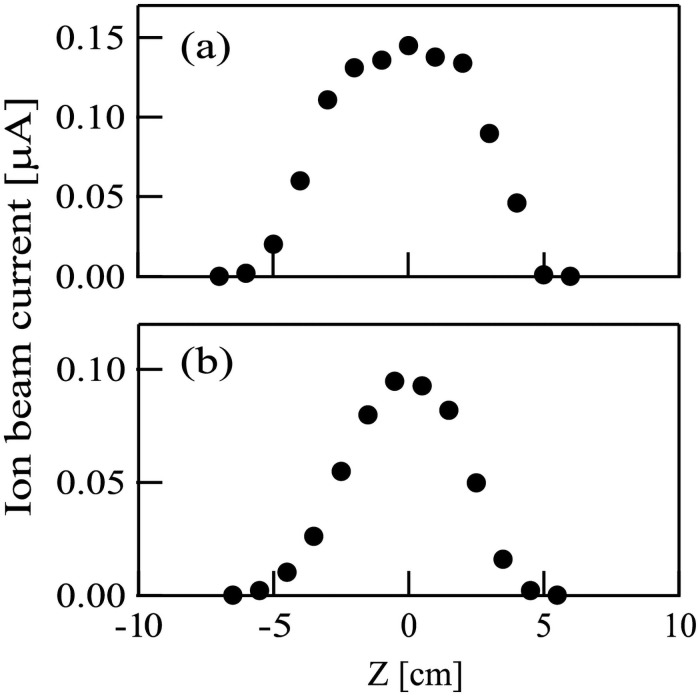
Typical intensity profiles for (a) Ar^+^ and (b) N^+^ ion beams. The ion energies of Ar^+^ and N^+^ ion beams were set at 100 eV. Horizontal axis represents the distance in the vertical direction.

The profiles of current intensities for (a) Ar^+^ and (b) N^+^ ion beams were measured using a molybdenum orifice plate. The orifice diameter was 2 mm. The profiles are shown in Fig 4a and 4b. The full widths at half maximum of the profiles were about (a) 7 mm and [Fig 4a] and (b) 5 mm [Fig 4b].

### Supply of hexamethyldisilazane to Si substrates with and without ion beams

Then, we prepared three samples, (a), (b), and (c). For the sample (a), a Si substrate was supplied with HMDS gas flow in conjunction with Ar^+^ ion beams. On the other hand, both HMDS and N^+^ ions were injected to a Si substrate for the sample (b). For the sample (c), a Si substrate was supplied with HMDS without any ion beams. For the samples (a)-(c), the duration of the experiments was 4h.

**Fig 5:**
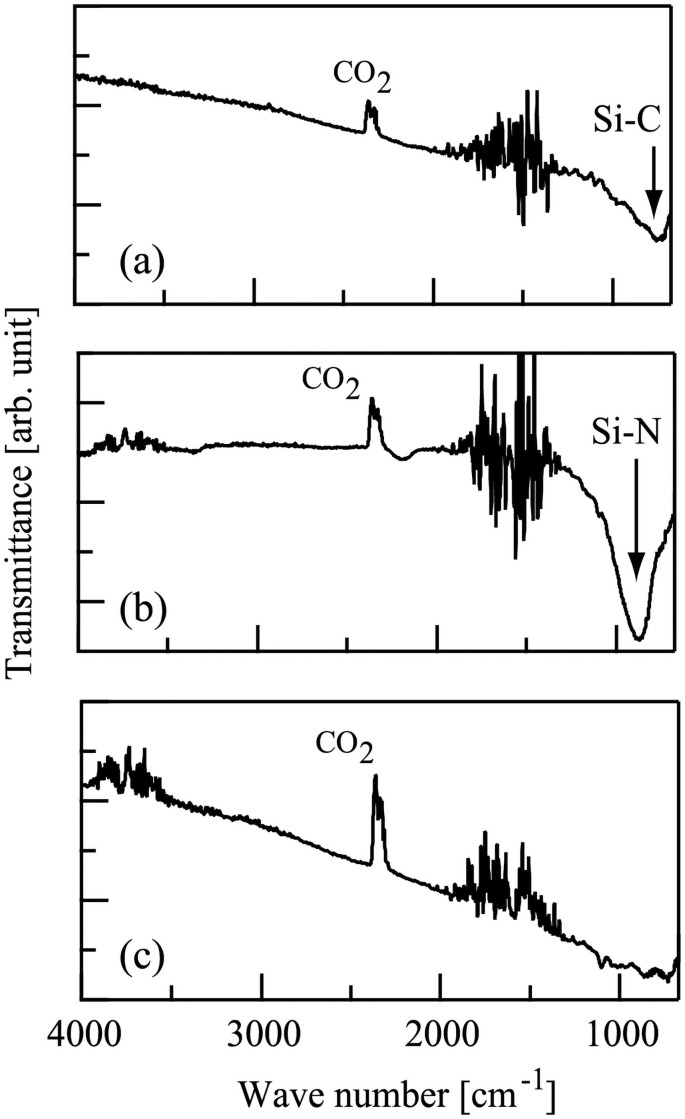
The Fourier-transform infrared spectra of films deposited on Si substrates following the injection of (a) Ar^+^ and (b) N^+^ ions in conjunction with hexamethyldisilazane. (c) The Fourier-transform infrared spectrum of a sample on a Si substrate following the supply of hexamethyldisilazane without ion beam irradiation. Substrate temperature during the deposition experiments was 540 °C. The duration of the deposition experiments was 4h.

We obtained FTIR spectra of these three samples in a transmission method using an FTIR spectrometer FT/IR-410 after the subtraction of the FTIR spectrum due to the Si substrate. The FTIR spectra of the samples (a) and (b) are shown in Fig 5a and 5b. The spectrum in Fig 5a has an absorption band at about 790 cm^-1^, showing that SiC film was deposited on the substrate [36]. On the other hand, the spectrum in Fig 5b has an absorption band due to Si-N [22], clearly indicating that SiN film was formed on the substrate. The FTIR spectrum of the sample (c) is shown in Fig 5c. On the contrary, Fig 5c shows no distinct peak, showing that almost no film formation occurred. We have also confirmed that no film formation was observed when substrates were irradiated with ion beams without HMDS. These results suggest that both of ion beam (Ar^+^ or N^+^) and HMDS were necessary for the formation of films on substrates. It was also noted that the peaks at about 1500 and 2400 cm^-1^ in Fig 5a–5c were attributed to water and carbon dioxide [37] in the ambient air inside the FT/IR-410 chamber.

### Low-energy Ar^+^ and N^+^ ion beam induced chemical vapor deposition using hexamethyldisilazane

The thicknesses of the films on the samples (a) and (b) were too thin to measure with a stylus profiler. Therefore, we prepared two new samples. In this case, the duration of the deposition experiment was 28 h, which was 7 times larger than the previous experiments. From now on, we will refer to these two samples as films (a) and (b). For the film (a), a Si substrate was injected with HMDS in conjunction with Ar^+^ ion beams. For the film (b), both of HMDS and N^+^ ions were injected to a Si substrate. The average currents of (a) Ar^+^ and (b) N^+^ ions were 1.4 and 2.0 μA, respectively. The total Ar^+^ and N^+^ ion doses were (a) 0.9x10^18^ and (b) 1.3x10^18^ ions.

Firstly, we measured the thicknesses of the films (a) and (b) with a stylus profiler P-15. The measurement showed that the thicknesses of the films (a) and (b) were about 20 and 200 nm, respectively. Although the doses of injected Ar^+^ and N^+^ ions were similar in the films (a) and (b), the film (b) was much thicker than the film (a).

**Fig 6:**
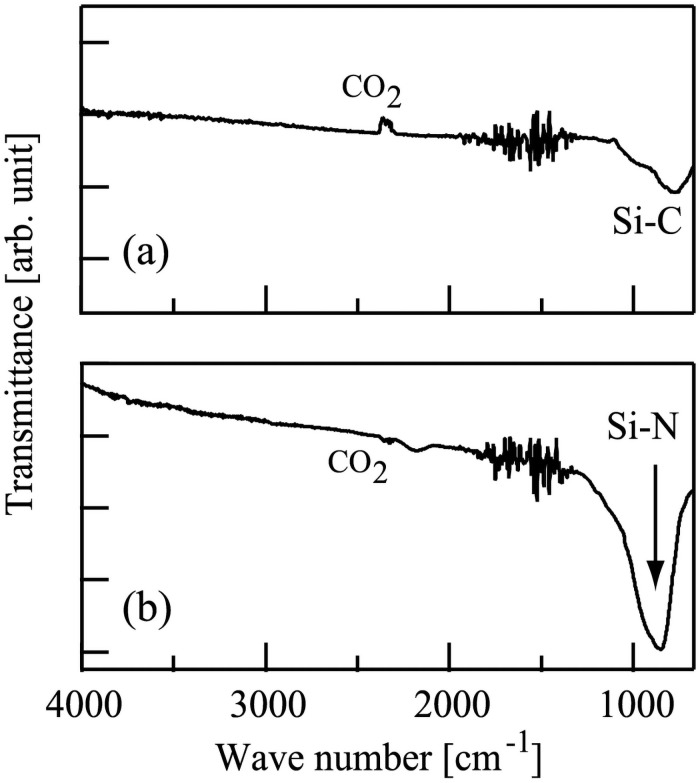
The Fourier-transform infrared spectra of films deposited on Si substrates following the injection of (a) Ar^+^ and (b) N^+^ ions in conjunction with hexamethyldisilazane. Substrate temperature during the deposition experiments was 540 °C. The duration of the deposition experiments was 28 h.

Then, we obtained FTIR spectra of these two films. The FTIR spectra of the films (a) and (b) are shown in Fig 6a and 6b. The spectra in Fig 6a and 6b are similar to those in Fig 5a and 5b, respectively.

**Fig 7:**
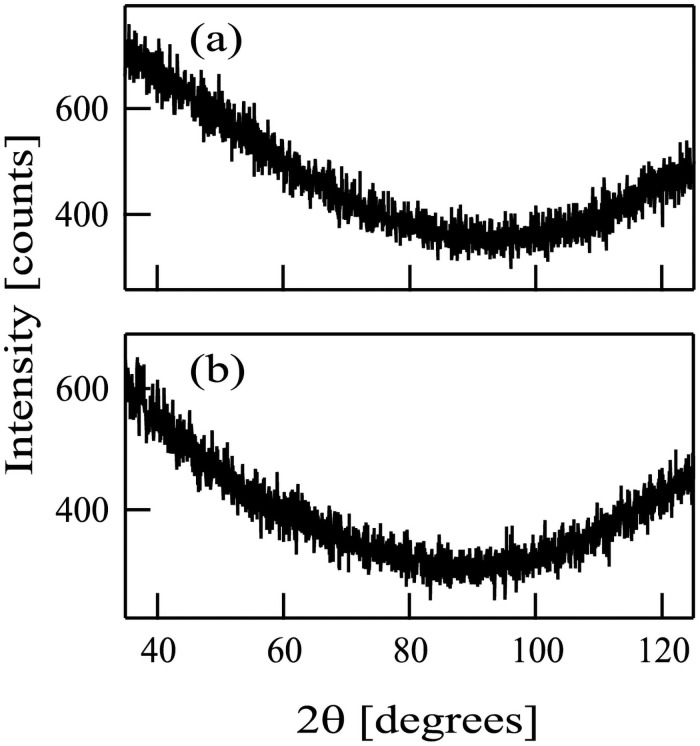
X-ray diffraction patterns of films deposited on Si substrates following the injection of (a) Ar^+^ and (b) N^+^ ions in conjunction with hexamethyldisilazane. Substrate temperature during the deposition experiments was 540 °C.

Subsequently, we analyzed the films (a) and (b) with an X-ray diffractometer RINT2200 using K_α1_ of Co. The XRD patterns (θ-2θ method) of the films (a) and (b) are shown in Fig 7a and 7b. No distinct peaks were observed in both Fig 7a and 7b, suggesting that there were no crystalline structures in the films (a) and (b) and both films were amorphous.

**Fig 8:**
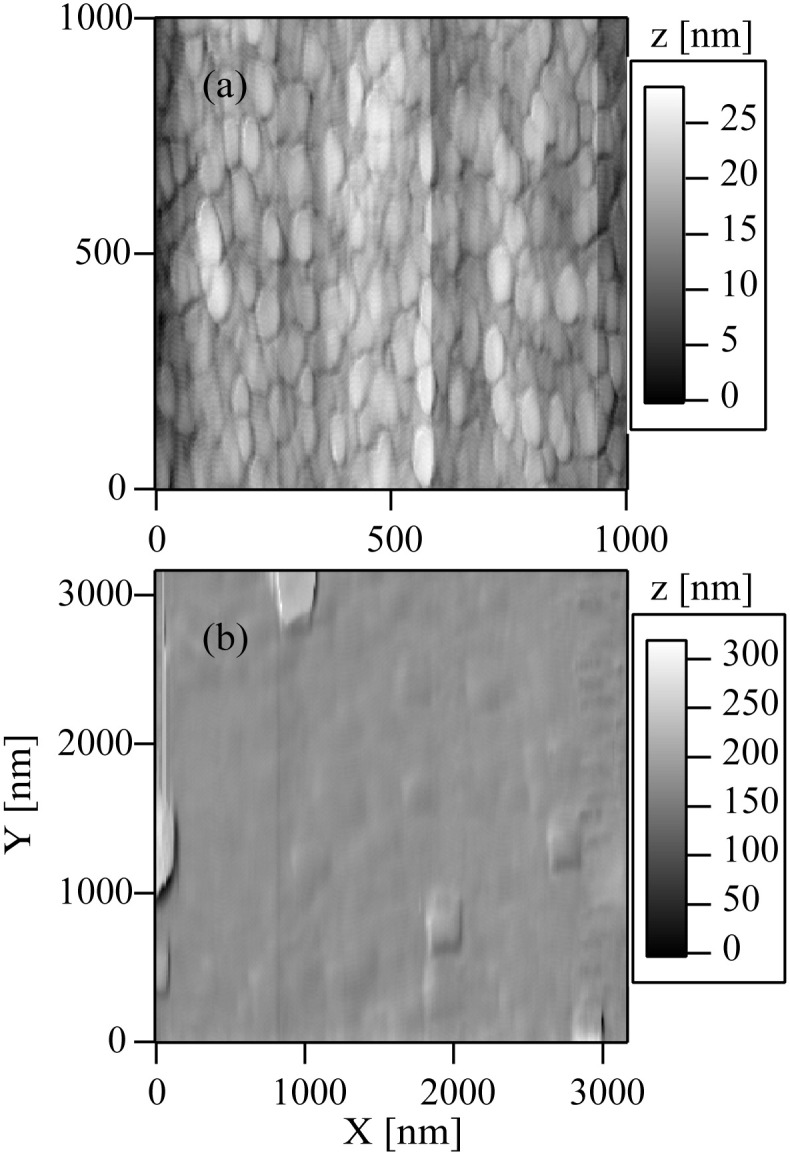
Atomic force microscopy images of films deposited when (a) Ar^+^ and (b) N^+^ ions were injected to Si substrates in conjunction with hexamethyldisilazane. Substrate temperature during the deposition experiments was 540 °C. The scale bars located in the right-hand side of the figures (a) and (b) indicate the height in units of nm.

Then, the films (a) and (b) were measured with JSTM-4200D. Fig 8a and 8b correspond to AFM images of the films (a) and (b). The root mean square surface roughness values for the films (a) and (b) were (a) 4 nm and (b) 14 nm, respectively.

**Fig 9:**
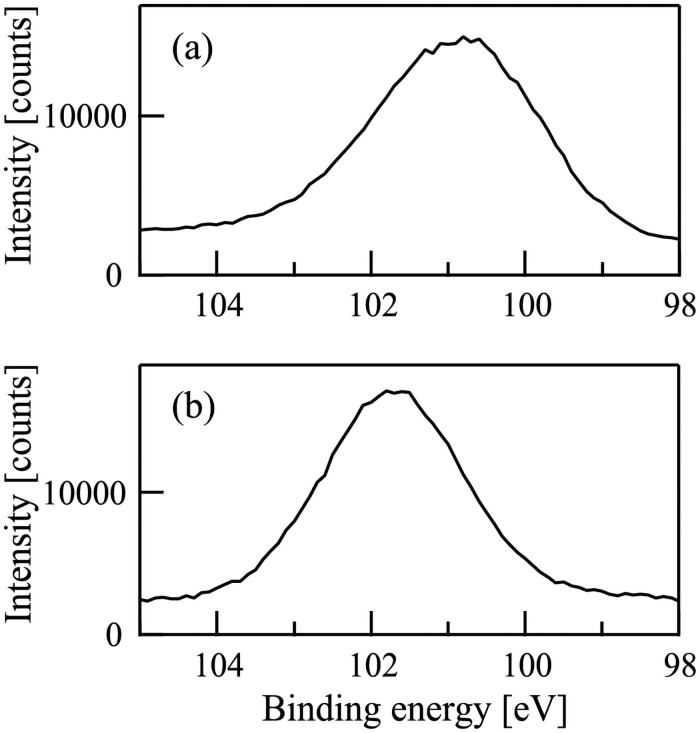
Si2p X-ray photoelectron spectroscopy spectra of films deposited following the injection of (a) Ar^+^ and (b) N^+^ ions to Si substrates in conjunction with hexamethyldisilazane. Substrate temperature during the deposition experiments was 540 °C.

Finally, the films (a) and (b) were analyzed with an XPS instrument AXIS-165x. Before the measurement, the films were etched with an Ar beam for removing contaminants. Fig 9a and 9b show the XPS spectra in the Si 2p region of the films (a) and (b). The peak of the binding energy in Fig 9a corresponds to that of SiC [38]. On the other hand, the peak binding energy in Fig 9b agrees with the presence of SiN [39].

An evaluation of the XPS data from the film (a) showed that the C to Si atomic ratio was 1.0. The XPS data also showed that a small amount of nitrogen atoms was included in the film (a). The nitrogen concentration was estimated to be 6%. On the other hand, the atomic concentration ratio of N to Si for the film (b) was evaluated to be 0.9. The XPS data showed that carbon atoms were included in the film (b). The carbon concentration of the film was estimated to be 10%.

## Discussion

In this study, FTIR and XPS assays of the film (a) showed that the nitrogen containing SiC film was formed on the substrate surface when Ar^+^ ions were injected in conjunction with HMDS. In previous papers, it was shown that nitrogen containing SiC films were available for thin overcoats for flexible media [40], materials with low dielectric constant [41], and materials of passivation layer [42].

We also found that the carbon containing SiN film was formed on the substrate when N^+^ ions were injected in conjunction with HMDS. There have been many papers to focus on SiN and SiCN film depositions with various experimental methods using HMDS [21–30]. These papers showed that the atomic concentration of Si, C, and N of films deposited using HMDS had a strong correlation with the experimental conditions. For example, it is necessary for the SiN deposition to investigate an optimum experimental condition, which can minimize the carbon elements to be incorporated in the film. The atomic concentration of carbon in the film (b) in this study seems to be reduced by increasing the dose of N^+^ ions injected. However, it is difficult to further increase the N^+^ ion beam intensity in our ion beam system.

SiN films can also be deposited on substrates using hexamethyldisiloxane and ammonia [43,44]. Ammonia should be, however, handled carefully in these deposition experiments because of being extremely harmful. On the other hand, the method presented in this paper has an advantage over other ordinary methods, all of which use ammonia, because both HMDS and nitrogen are less harmful.

## Conclusion

We here proposed an experimental methodology which made it possible to form films on substrates with an IBICVD method using HMDS.

We prepared three samples, (a), (b), and (c). For the sample (a), a Si substrate was supplied with HMDS gas flow in conjunction with Ar^+^ ion beams. On the other hand, both HMDS and N^+^ ions were simultaneously injected to a Si substrate for producing the sample (b). The sample (c) was produced on a Si substrate by providing HMDS without any ion beams. The Ar^+^ and N^+^ ion energies were both 101 eV. The temperature of the Si substrates was set at 540 °C. The duration of the experiments was 4h. After the experiments, the FTIR spectra of the samples (a) and (b) suggested that (a) SiC and (b) SiN films were actually deposited on the substrates. The FTIR spectrum of the sample (c), however, showed no distinctive peak, suggesting that no film deposition was observed when HMDS alone was supplied.

The thicknesses of the films on the samples (a) and (b) were too thin. Therefore, we prepared additional two new samples, films (a’) and (b’). In these cases, the duration of the deposition experiments was 28 h. For the film (a’), a Si substrate was injected with HMDS in conjunction with Ar^+^ ion beams. For the film (b’), both of HMDS and N^+^ ions were simultaneously injected to a Si substrate. The thicknesses of the films (a’) and (b’) were 20 and 200 nm, respectively. The FTIR spectra of the films (a’) and (b’) were similar to those of the samples (a) and (b), respectively. The XPS results of the film (a’) showed that a SiC film containing small amount of nitrogen was formed when Ar^+^ ions were injected in conjunction with HMDS. On the other hand, the XPS results of the film (b’) indicated that a SiN film involving carbon was formed.

We conclude that the IBICVD method using HMDS is useful for film formations.

## References

[1] Davis RF. Deposition, characterization, and device development in diamond, silicon carbide, and gallium nitride thin films. J. Vac. Sci. Technol. A 1993; 11: 829–837.

[2] Dimitrijev S, Jamet P. Advances in SiC power MOSFET technology. Microelectron. Reliab. 2003; 43: 225–233.

[3] Agnihotri OP, Jain SC, Poortmans J, Szlufcik J, Beaucarne G, Nijs J, et al. Advances in low temperature processing of silicon nitride based dielectrics and their applications in surface passivation and integrated optical devices. Semicond. Sci. Technol. 2000; 15: R29–R40.

[4] Matsunami H, Kimoto T. Step-controlled epitaxial growth of SiC: high quality homoepitaxy. Mater. Sci. Eng. R 1997; 20: 125–166.

[5] Matsumura H. Summary of research in NEDO Cat-CVD project in Japan. Thin Solid Films. 2001; 395: 1–11.

[6] Leinen D, Fernandez A, Espinos JP, Belderrain TR, Gonzalez-Elipe AR. Ion beam induced chemical vapor deposition for the preparation of thin film oxides. Thin Solid Films. 1994; 241: 198–201.

[7] Leinen D, Fernandez A, Espinos JP, Gonzalez-Elipe AR. Preparation of TiO_2_ and Al_2_O_3_ thin films by ion-beam induced chemical vapor deposition. Vacuum. 1994; 45: 1043–1045.

[8] Holgado JP, Perez-Sanchez M, Yubero F, Espinos JP, Gonzalez-Elipe AR. Corrosion resistant ZrO_2_ thin films prepared at room temperature by ion beam induced chemical vapor deposition. Surf. Coat. Technol. 2002; 151–152: 449–453.

[9] Matsui S. Focused-ion-beam deposition for 3-D nanostructure fabrication. Nucl. Instrum. Method Phys. Res. B 2007; 257: 758–764.

[10] Xu QY, Kageyama Y, Suzuki T. Ion-beam-induced chemical-vapor deposition of FePt and CoPt particles. J. Appl. Phys. 2005; 97: 10K308.

[11] Matsutani T, Asanuma T, Liu C, Kiuchi M, Takeuchi T. Ion beam-induced chemical vapor deposition with hexamethyldisilane for hydrogenated amorphous silicon carbide and silicon carbonitride films. Surf. Caot. Technol. 2003; 169–170: 624–627.

[12] Matsutani T, Asanuma T, Liu C, Kiuchi M, Takeuchi T. Deposition of SiO_2_ films by low-energy-ion-beam induced chemical vapor deposition using hexamethyldisiloxane. Surf. Coat. Technol. 2004; 177–178: 365–368.

[13] Yoshimura S, Sugimoto S, Murai K, Honjo K, Kiuchi M. Application of ion beam induced chemical vapor deposition for SiC film formation on Si substrates using methylsilane. e-J. Surf. Sci. Nanotechnol. 2015; 13: 174–178.

[14] Nation JL. A new method using hexamethyldisilazane for preparation of soft insect tissues for scanning electron microscopy. Stain Technol. 1983; 58: 347–351. doi: 10.3109/10520298309066811 6679126

[15] Braet F, de Zanger R, Wisse E. Drying cells for SEM, AFM and TEM by hexamethyldisilazane: a study on hepatic endothelical cells. J. Microscopy 1997; 186: 84–87. 10.1046/j.1365-2818.1997.1940755.x 9159923

[16] Bruynes CA, Jurriens TK. Catalysts for silylations with 1,1,1,3,3,3- hexamethyldisilazane. J. Org. Chem. 1982; 47: 3966–3969.

[17] Reddy PY, Kondo S, Toru T, Ueno Y. Lewis acid and hexamethyldisilazane-promoted efficient synthesis of N-alkyl- and N-arylimide derivatives. J. Org. Chem. 1997; 62: 2652–2654. doi: 10.1021/jo962202c 11671615

[18] Karimi B, Golshani B. Mild and highly efficient method for the silylation of alcohols using hexamethyldisilazane catalyzed by iodine under nearly neutral reaction conditions. J. Org. Chem. 2000; 65: 7228–7230. doi: 10.1021/jo005519s 11031058

[19] Azizi N, Saidi MR. Novel and efficient method for silylation of hydroxyl groups with hexamethyldisilazane (HMDS) under solvent-free and neutral conditions. Organometall. 2004; 23: 1457–1458.

[20] Lu H, Cheng J. Hexamethyldisilazane-mediated controlled polymerization of α-amino acid N-carboxyanhydrides. J. Am. Chem. Soc. 2007; 129: 14114–14115. doi: 10.1021/ja074961q 17963385

[21] Fainer NI, Rumyantsev Yu M, Kosinova ML, Yurjev GS, Maximovskii EA, Kuznetsov FA. The investigation of properties of silicon nitride films obtained by RPECVD from hexamethyldisilazane. Appl. Surf. Sci. 1997; 113–114: 614–617.

[22] Taguchi K, Yoshimoto M, Saraie J, Chayahara A, Horino Y. Dense structure of SiN_x_ films fabricated by radical beam deposition method using hexamethyldisilazane. Jpn. J. Appl. Phys. 2004; 43: L1403–L1405.

[23] Oyaidu T, Ogawa Y, Tsurumaki K, Ohdaira K, Matsumura H. Formation of gas barrier films by Cat-CVD method using organic silicon compounds. Thin Solid Films 2008; 516: 604–606.

[24] Kuo D-H, Yang D-G. Plasma-enhanced chemical vapor deposition of silicon carbonitride using hexamethyldisilazane and nitrogen. Thin Solid Films 2000; 374: 92–97.

[25] Fainer NI, Rumyantsev Yu M, Golubenko AN, Kosinova ML, Kuznetsov FA. Synthesis of nanocrystalline silicon carbonitride films by remote plasma enhanced chemical vapor deposition using the mixture of hexamethyldisilazane with helium and ammonia. J. Cryst. Growth 2003; 248: 175–179.

[26] Bulou S, Brizoual L Le, Miska P, de Poucques L, Hugon R, Belmahi M, et al. The influence of CH_4_ addition on composition, structure and optical characteristics of SiCN thin films deposited in a CH_4_/N_2_/Ar/ hexamethyldisilazane microwave plasma. Thin Solid Films 2011; 520: 245–250.

[27] Shayapov VR, Rumyantsev Yu M, Dzyuba AA, Ayupov BM, Fainer NI. Mechanical stresses in silicon carbonitride films obtained by PECVD from hexamethyldisilazane. Appl. Surf. Sci. 2013; 265: 385–388.

[28] Izumi A, Oda K. Deposition of SiCN films using organic liquid materials by HWCVD method. Thin Solid Films 2006; 501: 195–197.

[29] Demin VN, Smirnova TP, Borisov VO, Grachev GN, Smirnov AL, Khomyakov MN. Deposition of hard silicon carbonitride coatings from hexamethyldisilazane (HMDS) and HMDS-benzene vapors in laser plasma. J. Struct. Chem. 2020; 61: 1390–1397.

[30] Katamune Y, Mori H, Morishita F, Izumi A. Control of the chemical composition of silicon carbon nitride films formed from hexamethyldisilazane in H_2_/NH_3_ mixed gas atmospheres by hot-wire chemical vapor deposition. Thin Solid Films 2020; 695: 137750.

[31] Kim MT, Lee J. Characterization of amorphous SiC:H films deposited from hexamethyldisilazane. Thin Solid Films 1997; 303: 173–179.

[32] Yoshimura S, Sugimoto S, Takeuchi T, Murai K, Kiuchi M. Identification of fragment ions produced from hexamethyldisilazane and production of low-energy mass-selected fragment ion beam. Nucl. Instrum. Methods Phys. Res. B 2018; 430: 1–5.

[33] Chen L-Y, Hong F C-N. Surface tension studies of (Si, N)-containing diamond-like carbon films deposited by hexamethyldisilazane. Diamond Related Mater. 2003; 12: 968–973.

[34] Yoshimura S, Sugimoto S, Kiuchi M. Low-energy mass-selected ion beam production of fragments produced from hexamethyldisilane for SiC film formation, J. Appl. Phys. 2016; 119: 103302.

[35] Yoshimura S, Sugimoto S, Takeuchi T, Murai K, Kiuchi M. Low-energy mass-selected ion beam deposition of silicon carbide with Bernas-type ion source using methylsilane. AIP Adv. 2019; 9: 095051.

[36] Spitzer WG, Kleinman DA, Frosch CJ. Infrared properties of cubic silicon carbide films. Phys. Rev. 1959; 113: 133–136.

[37] Bruun SW, Kohler A, Adt I, Sockalingum GD, Manfait M, Martens H. Correcting attenuated total reflection-Fourier transform infrared specter for water and carbon dioxide. Appl. Spectr. 2006; 60: 1029–1039. 10.1366/000370206778397371 17002829

[38] Parrill TM, Chung YW. Surface analysis of cubic silicon carbide (001). Surf. Sci. 1991; 243: 96–112.

[39] Yu G, Edirisinghe MJ, Finch DS, Ralphi B, Parrick J. Synthesis of α-silicon nitride powder from a polymetric precursor. J. Eur. Cer. Soc. 1995; 15: 581–590.

[40] Scharf TW, Barnard JA. Nanotribology of ultrathin a: SiC/SiC-N overcoats using a depth sensing nanoindentation multiple sliding technique. Thin Solid Films 1997; 308–309: 340–344.

[41] Chiang C-C, Wu Z-C, Wu W-H, Chen M-C, Ko C-C, Chen H-P, et al. Physical and barrier properties of plasma enhanced chemical vapor deposition a-SiC:N:H films. Jpn. J. Appl. Phys. 2003; 42: 4489–4494.

[42] Huran J, Valovič A, Boháček P, Shvetsov VN, Kobzev AP, Borzakov SB, et al. The effect of neutron irradiation on the properties of SiC and SiC(N) layer prepared by plasma enhanced chemical vapor deposition. Appl. Surf. Sci. 2013; 269: 88–91.

[43] Jhansirani K, Dubey RS, More MA, Singh S. Deposition of silicon nitride films using chemical vapor deposition for photovoltaic applications. Results Phys. 2016; 6: 1059–1063.

[44] Xiao ZG, Mantei TD. Plasma-enhanced deposition of hard silicon nitride-like coatings from hexamethyldisiloxane and ammonia. Surf. Coat. Technol. 2003; 172: 184–188.

